# P05-10 Changes in mental health and physical activity patterns before and during the covid-19 pandemic in Swedish adolescents - a longitudinal study

**DOI:** 10.1093/eurpub/ckac095.077

**Published:** 2022-08-29

**Authors:** Gisela Nyberg, Karin Kjellenberg, Björg Helgadóttir, Örjan Ekblom

**Affiliations:** 1 Department of Sport Science, The Swedish School of Sport and Health Sciences, Stockholm, Sweden; 2 Department of Global Public Health, Karolinska Institutet, Stockholm, Sweden

**Keywords:** Sedentary, accelerometry, health-related quality of life, psychosomatic health

## Abstract

**Background:**

The covid-19 pandemic has had a large impact on the daily lives of adolescents, even in Sweden where the restrictions were relatively mild. The aim of this study was to examine if there had been a change in mental health outcomes and if these changes were related to changes in physical activity patterns before and during the pandemic.

**Methods:**

In this longitudinal study, data were collected in the autumn 2019 and in follow-up measurements in the spring 2021. Physical activity and sedentary time were measured for seven consecutive days by accelerometry (Actigraph). The mental health outcomes, health-related quality of life (HRQoL) and psychosomatic health were measured with questionnaires (KIDSCREEN-10 and PSP). ANCOVA analyses were applied to estimate the associations between change in physical activity patterns and mental health outcomes.

**Results:**

In total, 585 boys (45%) and girls (55%), aged 13-14 years (baseline) from 34 schools around Stockholm, were included in the study. Between 2019-2021 there was a decrease in HRQoL (p > 0.001) and increase in psychosomatic problems (p > 0.001) among both boys and girls. There was a significant positive relationship between change in MVPA and change in HRQoL (β = 0.02, CI: 0.00, 0.05).

**Conclusions:**

The results suggest that the COVID-19 pandemic has impaired the mental health of Swedish adolescents but increased physical activity was related to positive changes in the mental health outcome HRQoL.

Funding: The Public Health Authority and Skandia

